# Relationship between FDM 3D Printing Parameters Study: Parameter Optimization for Lower Defects

**DOI:** 10.3390/polym13132190

**Published:** 2021-06-30

**Authors:** Patrich Ferretti, Christian Leon-Cardenas, Gian Maria Santi, Merve Sali, Elisa Ciotti, Leonardo Frizziero, Giampiero Donnici, Alfredo Liverani

**Affiliations:** Department of Industrial Engineering, Alma Mater Studiorum—University of Bologna, I-40136 Bologna, Italy; patrich.ferretti2@unibo.it (P.F.); christian.leon2@unibo.it (C.L.-C.); gianmaria.santi2@unibo.it (G.M.S.); merve.sali2@unibo.it (M.S.); elisa.ciotti@studio.unibo.it (E.C.); giampiero.donnici@unibo.it (G.D.); alfredo.liverani@unibo.it (A.L.)

**Keywords:** optimized FDM, defects, printing parameters, optimization, void occurrence

## Abstract

Technology evolution and wide research attention on 3D printing efficiency and processes have given the prompt need to reach an understanding about each technique’s prowess to deliver superior quality levels whilst showing an economical and process viability to become mainstream. Studies in the field have struggled to predict the singularities that arise during most Fused Deposition Modeling (FDM) practices; therefore, diverse individual description of the parameters have been performed, but a relationship study between them has not yet assessed. The proposed study lays the main defects caused by a selection of printing parameters which might vary layer slicing, then influencing the defect rate. Subsequently, the chosen technique for optimization is presented, with evidence of its application viability that suggests that a quality advance would be gathered with such. The results would help in making the FDM process become a reliable process that could also be used for industry manufacturing besides prototyping purposes.

## 1. Introduction

The Fused Filament Fabrication (FFF) process was first introduced under the name of FDM^®^ in the 1990s, patented as a moldless, fabrication method for three-dimensional solid objects (US Patent No. 5,738,817). Nowadays, it is among the most popular additive manufacturing techniques because it offers a versatile choice of thermoplastic materials [1]. It consists of a continuous process of depositing successive layers, from the bottom to the top by heating and extruding a filament [2], therefore, building a three-dimensional solid object having complex shapes, as reported by the studies of SAVU et al. [3], Mahamood et al. [4], and Brian et al. [5]. The research by Tofail et al. [6] stated that FDM can build fully functional parts of a product.

In addition, a potential cost-effective solution for small-scale components can be found in the metal-fused filament fabrication (FFF) process, since regular desktop FFF printers could be used to create metal-sourced objects [7].

### 1.1. FDM 3D Printing

Additive manufacturing (AM) or 3D printing technology is the definition of a methodology that can produce complex, irregular shaped three-dimensional (3D) which would be more time- and resource-consuming if used traditional machining methods for its manufacturing. Producing parts in small lots at a fast speed without a mold.

Achieving a better understanding in FDM processing promises to solve cost problems of other non-conventional FDM methodologies like SLS (Selective Laser Sintering), Poliget (Stratasys, Object), SLA (Stereolithography), DLP (Digital Light Processing), and MSLA (Masked Stereolithography) as reviewed by [8,9,10,11,12] that had shown to reach better quality than FDM, but could be more expensive to apply due to the need to use a more specialized equipment, the type of polymers available [13] for each method, and the need to add other components like resins that will be important to reach desired mechanical characteristics.

### 1.2. Defects in 3D Printing

**Figure 1:**
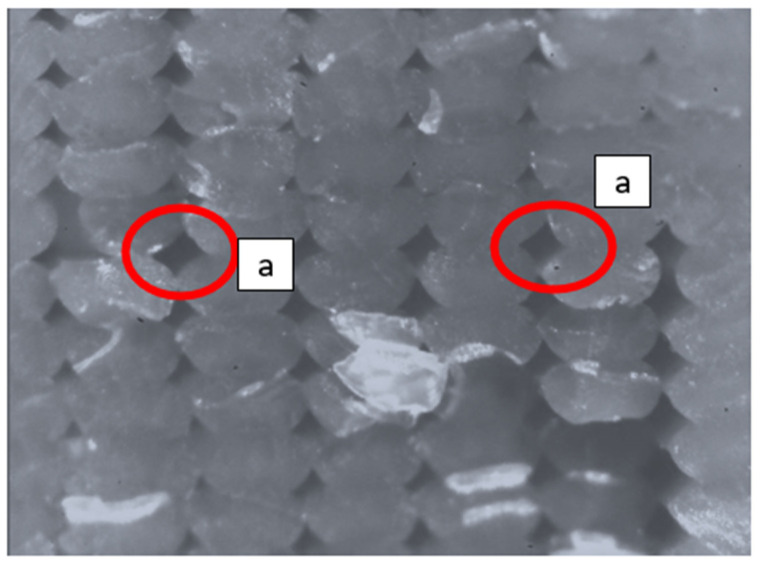
Defect appearance on a PLA specimen: (**a**) material voids between adjacent lines.

Defects play a key role in 3D printing since they are responsible for the reduction in mechanical properties with respect to injection molded parts. As suggested in [14], the presence of pores/voids in the 3D printed structure leads to a decrease in the final density of the specimen and depends on the printing parameter. In addition, in the FDM printing process, it is necessary to optimize and reduce the presence of voids in the structure because, even if the defects undergo a sintering process, they decrease in size, but still remain present in the structure. Moreover, some pores appear due to the chosen printing strategy (i.e., places where two perimeters joined or where the infill started and jointed). This alignment of pores was observed in CT (Computed Tomography) scans of specimens produced by FFF with other highly filled filaments [15], and they can lead to weaker mechanical properties. This phenomenon could be seen in Figure 1.

A deep knowledge of FFF process parameters is required to obtain objects with improved mechanical properties (i.e., tensile strength, compressive strength, etc.) [16]. Moreover, it was observed that FFF 3D printing introduces anisotropic behavior to the manufactured part by means of gaps that could reduce its tensile strength, both in modulus and in its failure [17,18,19,20,21,22].

### 1.3. Volumetric Flow Rate and Density

**Figure 2:**
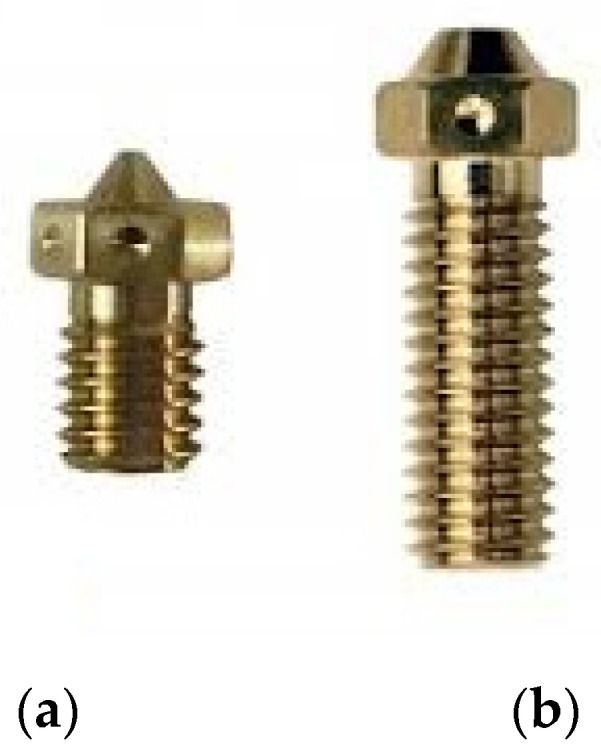
Nozzle length difference according to (**a**) lower volumetric flow rate, (**b**) higher volumetric flow rate.

The density of the material that is coming off from the nozzle is the result of a series of parameters all inextricably linked together. In order to identify the complexity of the problem, an understanding about the deformation field due to the surface tension and the Laplace pressure difference, as reported by Liu et al. [23], and the possibility that the liquid/vapor interface is pinned on the microstructures, is needed to have an idea about the behavior of the molten material at high temperature [24]. In the ideal case of constant extrusion speed over time, the density of the melt coming out of the nozzle is related to the set extrusion temperature and the nozzle material and length. In fact, the nozzle (in most cases) also acts as a melting chamber in which the thermoplastic is brought to a sufficient density to be extruded. The heat transfer between heated block and material depends on the thermal resistance value of the nozzle. Furthermore, the geometry of the nozzle and heated-block itself influences the final density of the extruded filament a lot—for example, the differences between E3D v6 and E3D volcano nozzles as shown in Figure 2.

Constraining the extrusion at constant speed is not a real hypothesis, as the extrusion speed varies over time, even within the same layer, although the speed set in the slicer is the same for every sector of the layer (shell, infill, etc.), there are still variations in speed due to the changes of direction and the fact that, for every line that is not continuous, the extruder starts from zero speed, accelerates, reaches, if possible, the set speed, decelerates, and stops. These variations obviously also affect the extrusion speed and consequently the time the filament has to be heated inside the fusing chamber, and therefore there are small variations in the density of the extruded material, previously studied by Pan et al. [25].

It should be noted that, even varying the layer height, keeping the extrusion temperature constant, results in a variation of the density of the extruded material. Increasing the layer height will require extrusion of a larger quantity of material (higher volumetric flow rate), and, consequently, to have the same degree of adhesion, the extrusion temperature will have to be increased. Therefore, if it is decided to carry out tests on the best layer height value, the need to take into account the volumetric flow value, and to adjust the temperature accordingly. Interlayer bonding quality is therefore a result of the temperature of the extruded material.

*Volumetric Flow Rate*: depends on a large number of factors, it depends directly on the actual print speed, width (at width increase, increase the amount of extruded material), and layer height (greater and higher the amount of extruded material in the unit of time), as suggested by Percoco et al. [16], the difference of which could be seen in Figure 2.

*Delta Temperature*: the extrusion temperature, together with the “environment” temperature, determine the actual density of the newly deposited material.

*Nozzle Material*: Thermal conductivity of steel (23 W/m·K), or copper (330 W/m·K).

By giving a wider desertion about the density explanation performed by Pan et al. **[25]**, density is consequently a key factor, together with the temperature of the newly extruded material that allows for achieving excellent adhesion between one layer and the one afterwards. In order to simplify this model and make it as general as possible, the effect of density will be incorporated as a correction factor to the main theoretical model.

### 1.4. A Model for Defect Analysis in FDM Printing

The model arises from the need to understand the influence of the main printing parameters on the volume of defects present in the workpiece, in order to make this analysis as general as possible in a way that it can be used in common polymers for application in FDM process, but also for MetFDM.

The model focuses on the identification of type of defects and the theoretical volume of those. The model aims to explore the effect of changing nozzle diameter, changing width, number of shell lines, slicing angle on the single layer, and changing part size, keeping the above parameters constant.

**Figure 3:**
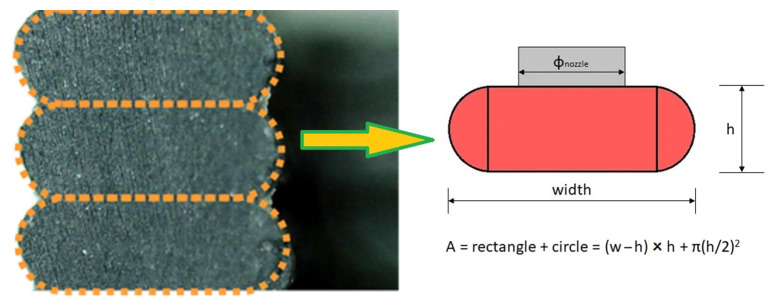
Printing line geometry characterization.

The starting point is the analysis of the shape of a single line and its parameterization. It was verified that the model presented by Slic3r [26] and then taken up by PrusaSlicer [27] and reported in Figure 3 is valid as a simplification. In fact, each line is designed with a geometry that combines two semicircles and a rectangle. This geometry is in fact the same one that uses the slicer internally to generate the toolpath.

## 2. Materials and Methods

### 2.1. Model Construction

#### 2.1.1. Geometry

The geometry chosen to analyze the model is a solid cube (infill set at 100%), the dimensions of which are set at 30 × 30 × 30 in order to analyze the effect of the various printing parameters; width and length are changed only if the effect of the component size on the defect volume is analyzed.

#### 2.1.2. Defect Instances

The first hypothesis of this model is that each line touches (in section) the adjacent line only at one point. This hypothesis allows us to get into a “standard” state, which is in fact also the way the slicer creates the toolpath, imagining that each line touches the adjacent line along a line. The infill is set to 100% and the selected infill type is “lines”. The fact that in reality it is possible for lines to have a non-point contact area will be considered later, as it is in fact an “improvement” over the point contact condition.

Therefore, four types of defects were identified. Defects refer to the presence of gaps in the structure that are not generated randomly, but depend on how the material is placed. This type of defect is then repeated and, if the conditions during printing do not change (constant extrusion temperature, constant ambient temperature, no speed changes during printing, etc.), this type of defect is repeated on each layer.

(A) Defect that considers the volume of missing material compared to a perfectly flat surface, similar to a surface made with a traditional manufacturing process like injection molding, for example.

(B) Defects that appear between a shell line and the adjacent shell line; if there are more than two contour lines, this defect ponders the total volume of the voids.

**Figure 4:**
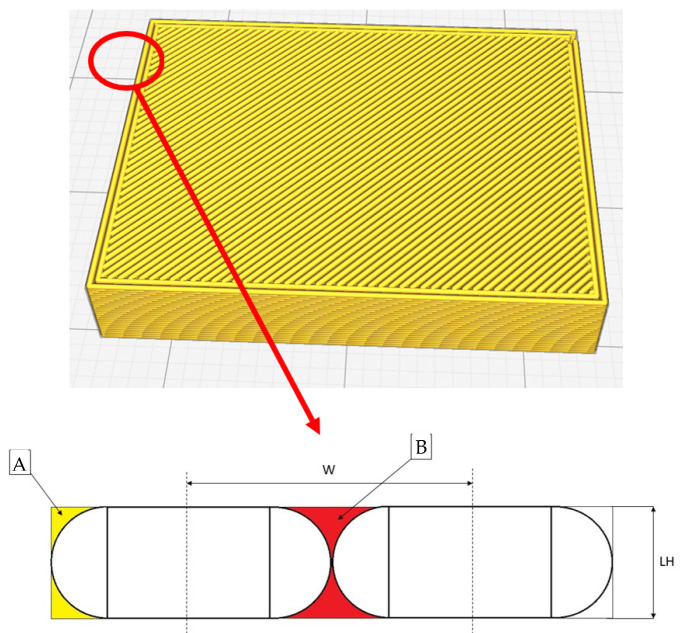
Defects (A,B).

**Figure 5:**
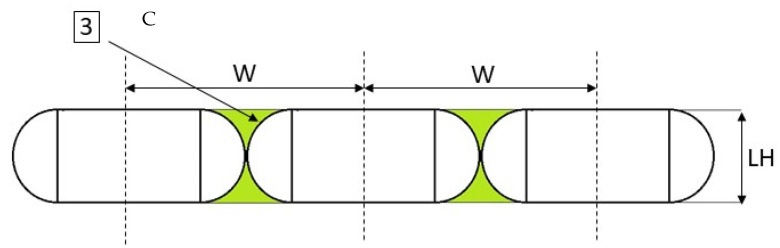
Defect (C).

**Figure 6:**
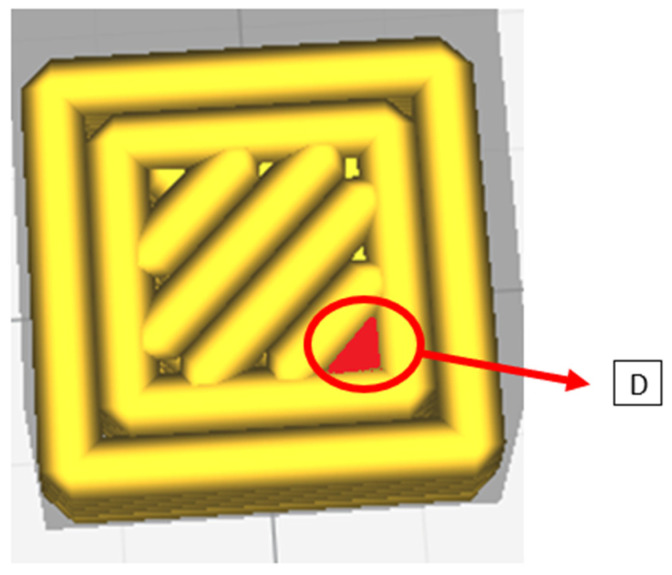
Defect (D).

Details of such defects could be seen in Figure 4.

(C) From a purely geometric point of view, there are no differences in the geometry of defects B and C, defect C however refers to the lines of the infill (Figure 5).

(D) Defect D, as seen in Figure 6, a defect that takes into account the formation of empty areas (without extruded material) due to the fact that the number of lines in the infill is approximated by default.

**Figure 7:**
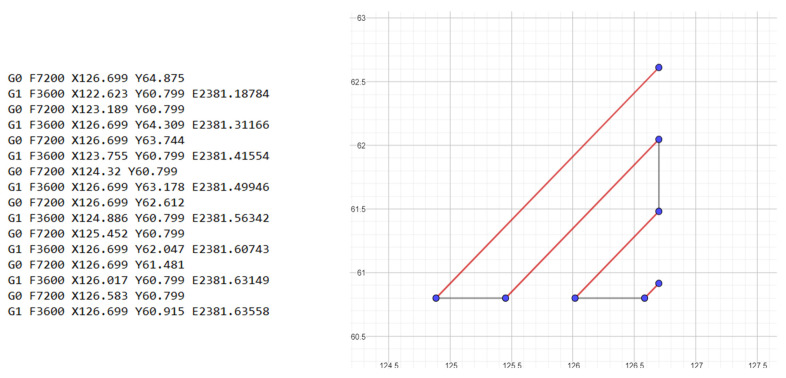
Example of a printing gcode (**left**); last 8 lines of gcode drawn in geogebra (**right**); in red: the extrusion moving path, in gray: the shift movement of a single printer head.

After identifying the type of defects and using a spreadsheet, an algorithm was created to calculate the total volume of defects. To calculate the volume of defects type A and B, it was sufficient to know the width of each line and the initial size of the cube; for defects C and D, it was necessary to know the length of each line. The verification of the algorithm as seen in Figure 7 was done using gcode generated by Cura software, and then plotting the various points and lines present in the gcode.

#### 2.1.3. Algorithm Parameters

The parameters taken into account for this study are defined by the total geometry of the model, geometry of the extruded, molten material and the number of contour lines that it would take to form the part. Details for parameters of input (Table 1) and output (Table 2).

## 3. Results

### 3.1. Parameter Influence

#### 3.1.1. Influence of Shell Number

**Figure 8:**
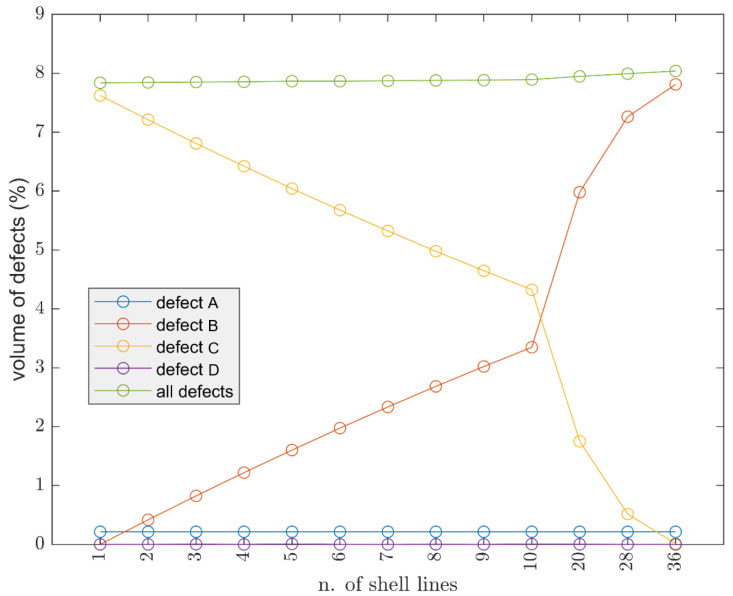
Influence of the number of shell (contour) lines on 30 × 30 × 30 mm cube, nozzle 0.4 mm, line width 0.4 mm, layer height 0.15 mm, raster angle 45°.

A number of essays were performed by printing a cube of 30 × 30 × 30 mm, and keeping constant layer height (0.15 mm) and width (0.4 mm); Figure 8 shows the trial performed by keeping values for raster angle of the infill at 45° and increasing the value of shell lines. This results in the total volume of defects increasing as the number of contour lines increases. Since the layer height is constant, defects type A and D remained constant. By varying type B and type C, the variation of the latter leads to a slight increase in the total volume of defects.

Afterwhile, it can be said that it is good to reduce the number of shell lines as increasing them does not bring any visible advantage. Furthermore, as theoretically the effect is minimal, in real terms, it presents some challenges, as the contour lines do not have a raster angle, and this can result in adhesion issues afterwards.

#### 3.1.2. Influence of Width

The width represents the average distance between a line and the next. Increasing the width, as shown in the Figure 8, the total volume of defects can be reduced. The lower limit for the value of the width is usually set equal to the diameter of the nozzle, and there is the possibility to reduce the value slightly (e.g., when you have to make thin walls, not multiples of the width, in order to avoid gaps in the printed part), in all other cases, it is advantageous to increase the width. The maximum attainable value is limited by two factors:

- the diameter of the flat area of the nozzle;
- the density of the extruded material.

**Figure 9:**
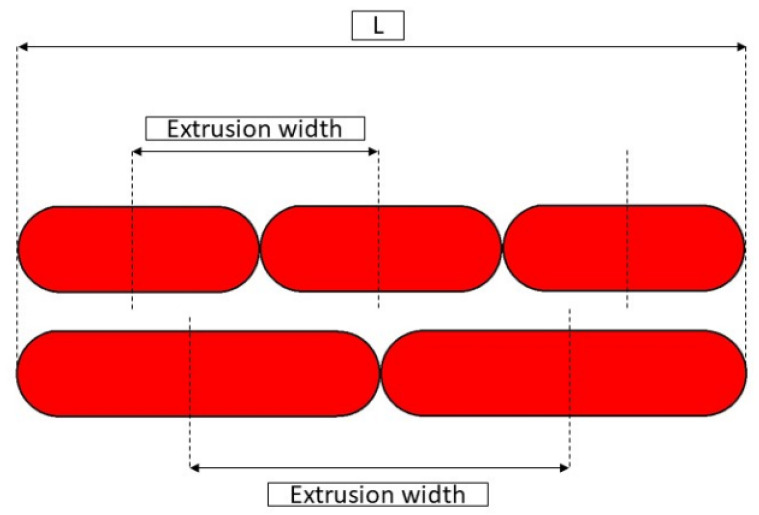
Lower extrusion width (on top) and higher extrusion width (on bottom) compared for a given dimension L.

**Figure 10:**
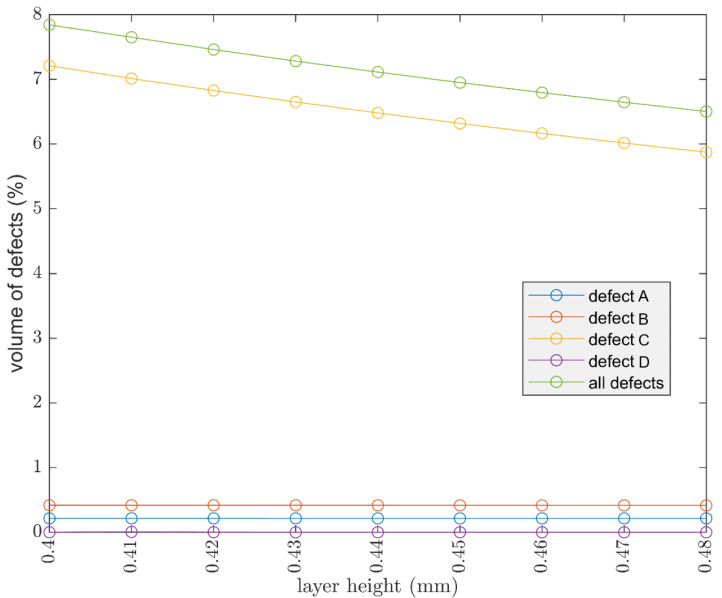
Influence of width (considering 2 shell (contour) lines, raster angle 45°, nozzle 0.4 mm, layer height 0.15 mm).

For the first point, it is essential that all extruded material is contained below the nozzle; otherwise, defects may occur on the print surface. The density of the extruded material is crucial, as the material is not simply deposited but is also subjected to a shear stress against the surface of the nozzle and the layer underneath. This results in a backpressure inside the nozzle which increases as the extrusion temperature decreases (extruded filament density). It should also be noted that increasing the width increases the volumetric flow rate because it increases the amount of material deposited in the unit of time. The risk of using temperatures that are too low is that of generating filament stripping or a loss of E steps. Increasing the width is therefore a very powerful tool but is needed to check the print parameters carefully. Results of width influence could be seen in Figure 9 and Figure 10.

#### 3.1.3. Layer Height

It was chosen to keep the width constant and equal to the nozzle diameter, raster angle at 45°, two shell lines.

Decreasing the layer height is an effective way to reduce the volume of defects, and it is very interesting to evaluate what happens by changing not only the layer height, but also the size of the nozzle.

**Figure 11:**
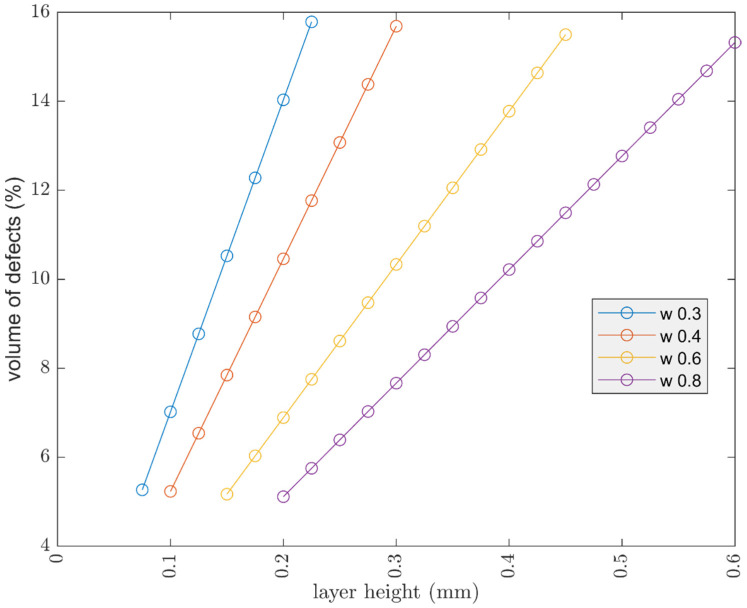
Different nozzle diameter compared at different layer heights; the width is equal to nozzle diameter for each of those.

The same cube of 30 × 30 × 30 is kept, but the diameter of the nozzle is increased and therefore the value of width is increased. Valid values for the layer height are a range between 15 and 75% of the value of the considered nozzle. It is interesting to see how the ability to use a larger nozzle can allow you to print at a higher layer height and also reduce the number of defects. This is possible because increasing the diameter of the nozzle also increases the width. Figure 11 outlines the effect of layer height by considering different nozzle diameters.

#### 3.1.4. Workpiece Size

Considering a parallelepiped with a square base and fixed height of 30 mm, the aim was to evaluate the theoretical trend of the defects volume, by increasing and decreasing the section. The cube parameters are: nozzle 0.4 mm, width 0.4 mm, two shell lines, and layer height of 0.15 mm.

**Figure 12:**
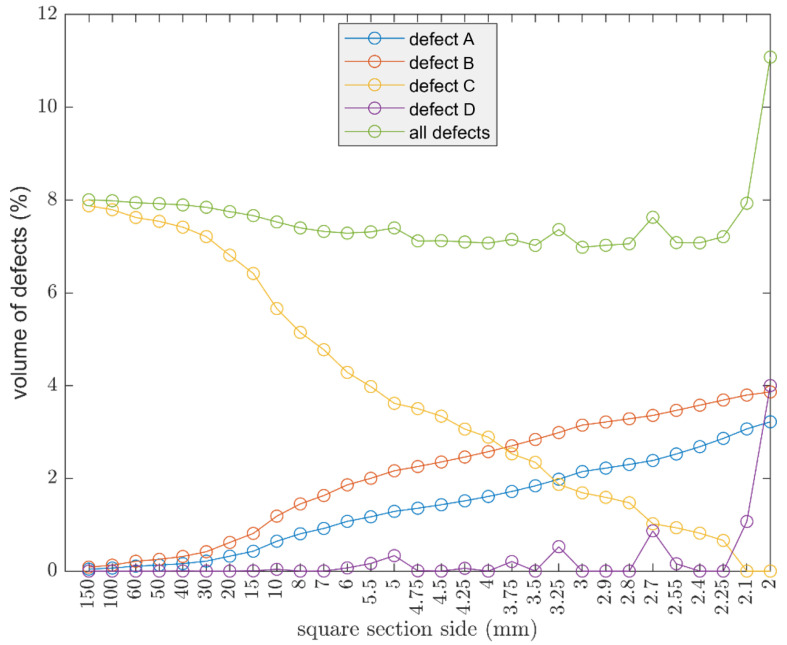
Influence of geometric dimension on volume of defects, layer height 0.15 mm, width 0.4 mm, 2 shell (contour) lines, raster angle 45°.

Displayed in Figure 12, by increasing the section, the volume of defects increased as expected. The trend begins to undergo important variations when the section decreases. Finally, at very small sizes, 2 mm, a peak in the volume of defects can be seen. The oscillations are due to defect D, seen in Figure 6, which becomes more important as the cross section decreases. Defect D arises from the approximation, by defect, of the number of internal lines, hence the behavior is oscillatory.

**Figure 13:**
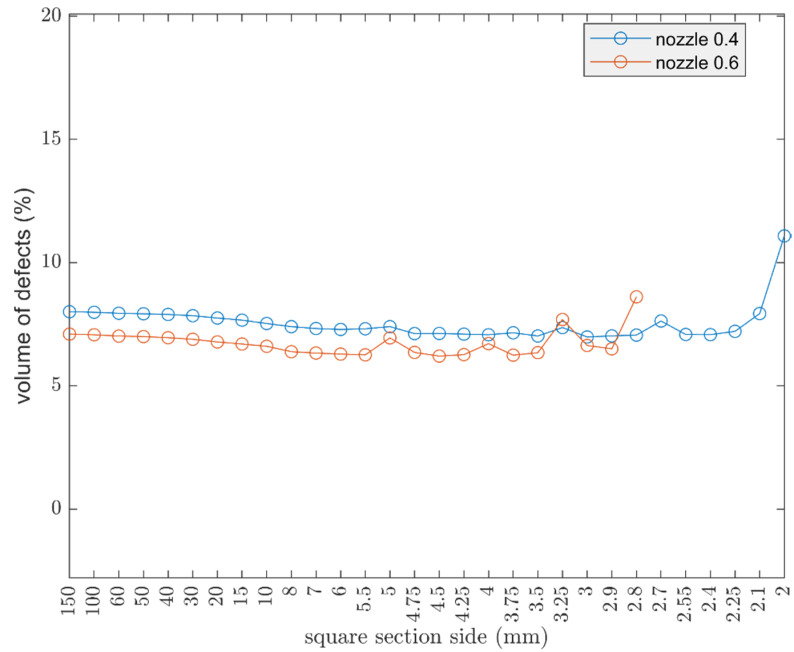
Comparison on the influences of the geometry considering the 0.4 mm and 0.6 mm nozzle; in the case of a 0.4 mm nozzle, the layer height is 0.15 mm, width 0.4 mm; in the case of a 0.6 mm nozzle, the layer height is 0.2 mm and width 0.6 mm.

Afterwards, the use of a larger nozzle size, such as 0.6, allows for a reduction of defects as already noted, seen in Figure 11, but the oscillatory behavior becomes evident at higher values of the cross section, as shown in Figure 13, with respect to the counterpart with a smaller nozzle.

## 4. Discussion

The mathematical model allows for evaluating the theoretical behavior, but, in order to take into account the effect of density (temperature, flowrate, etc.), and the possibility to have a defects reduction related to printing parameters’ optimization, the following formulation is proposed:

V% = K_A_V_A_ + K_B_V_B_ + K_C_V_C_ + K_D_V_D_

V% is the total volume in percentage of the occurrence of defects, the parameters K_A_, K_B_, K_C_, and K_D_ allow for adjusting the theoretical model to the real result.

These values can be obtained experimentally, by observing the layer in sections, through a common microscope and then performing an image analysis.

K_A_ and V_A_: the first element of the equation is related to defects that are present on the surface of the workpiece, this term can also be related to several aspects including surface roughness which is directly related to layer height and the staircase effect. It is difficult to obtain a reduction of this term by changing only the slicing parameter, but it is possible to obtain a KA value lower than 1 for some materials (e.g., Polymaker PVB) related to a chemical smoothing.

K_B_ and K_C_ take into account the same type of defect, one in shell lines and the other in infill lines.

However, K_B_ and K_C_ are not identical and often have different values.

Shell lines are always stacked on top of each other with a 0° raster angle. This leads to a high difficulty in reducing the defects between these lines.

Experimental evidence showed a maximum reduction of around 80% and the value of parameter KB in the range K_B_: 1–0.8.

For K_C_ instead, related to the possibility to have a raster angle for the infill lines between different layers and related to a proper selection of printing parameters, a greater reduction of the gaps between lines is possible. The value of K_C_ parameter is in the range between 0 and 0.9.

K_D_V_D_ is related to the presence of voids in the infill, and a reduction of these defects selecting the option “fill small gaps” in the slicer is possible.

### Optimization Scheme and Reduction for KC

**Figure 14:**
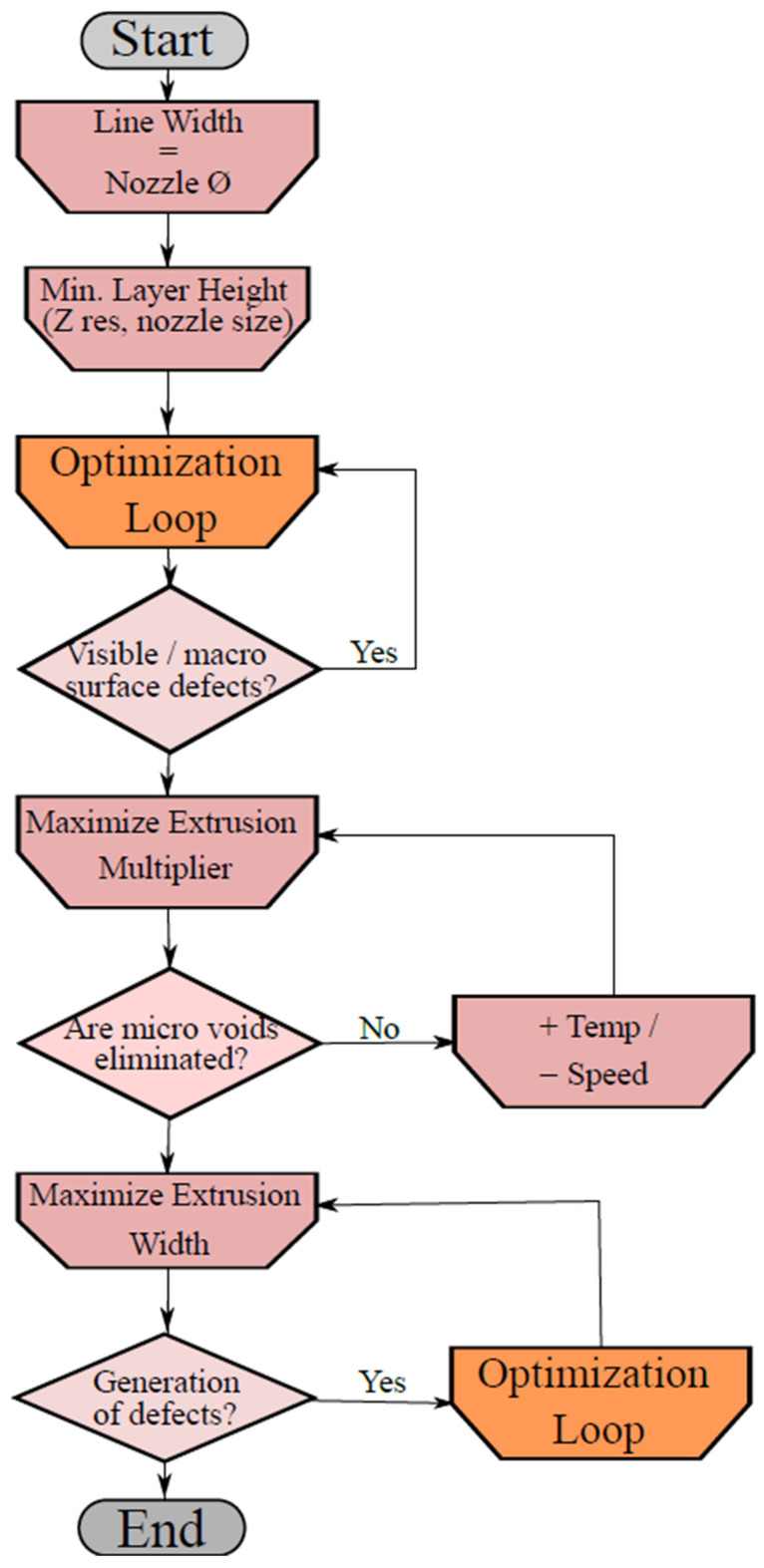
Flow chart of the optimization process.

**Figure 15:**
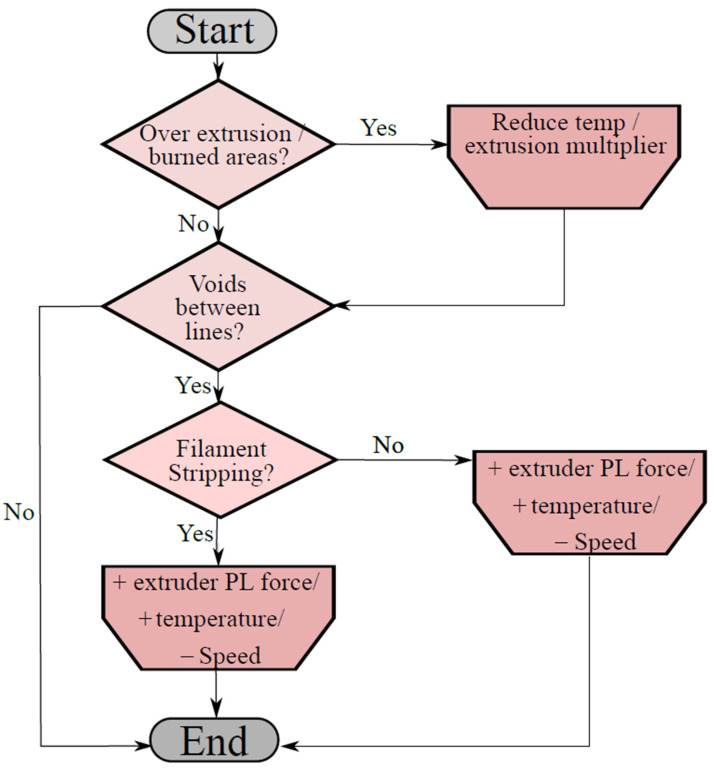
Optimization process loop cycle.

The proposed optimization procedure is reported in Figure 14 and Figure 15 that allows for reducing the values of KB and KC, and, consequently, to the total number of voids/defects existing on the specimen. The procedure allows for finding the best performing printing parameters given a specific 3D printer device and filament typology; the correct choice of printing parameters would guarantee optimal mechanical properties of the printed elements with zero internal voids.

**Figure 16:**
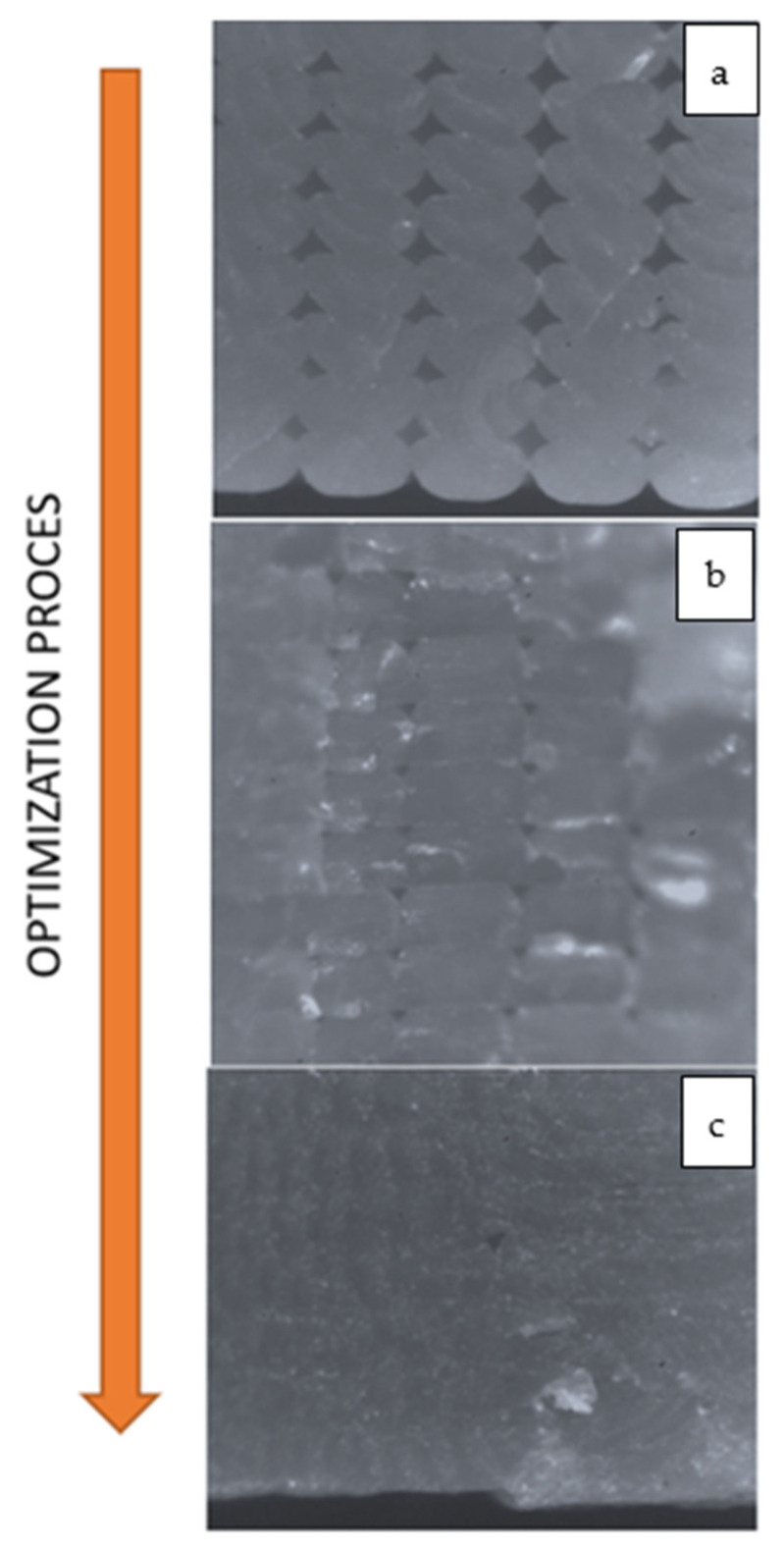
Printing quality on the microscope (20×): (**a**) not optimized; (**b**) increasing performance; and (**c**) fully optimized.

This optimization process starts by using the recommended printing settings given by the filament producer. At a first stage, the width must be set equal to the nozzle dimension. Afterwards, the minimization of the layer height is performed according to the nozzle diameter and the minimum resolution value of the printer. The first optimization loop cycle, seen in Figure 15, is needed to remove the macro defects on the printed surface of the part. An additional process is focused on removing defects B and C from the part. In Figure 16, it is possible to see the result of the proposed optimization loop on a 3D printed PLA specimen.

## 5. Conclusions

A number of different printing trials demonstrated that a variation in the slicing parameter has a direct effect on the appearance on the four different defect occurrence types. Furthermore, a reduction of the defect volume is proven to be feasible by means of a modification of input printing parameters like layer and line dimensions, overall number of shells, as well as filament-specific printing parameters like the extrusion multiplier and temperature. Additional reduction of defects is possible by means of the application of the proposed optimization methodology that would allow for gathering a correct value of printing settings. Furthermore, the volume of defects implies that mechanical characteristics of the material would also be compromised because of a poor choice of printing parameters for a given 3D printer and filament type. Internal material consistency is not guaranteed, making the material susceptible to developing failure due to a poor internal stability.

This approach and the proposed formula made it possible to make a valuable comparison between the volume of defects existing inside the specimen and the effect of the optimization procedure in the reduction of such voids, ending up with an internally quasi-isotropic structure that would mechanically sustain stresses in a similar way to the material manufactured from a regular-sourced procedure like injection molding.

This work therefore represents a step forward in order to turn FDM technology processes into the mainstream production of components with higher mechanical properties and making this process feasible for creating structural-applicable parts and not just for aesthetics or prototyping.

## Figures and Tables

**Table 1:** Algorithm main input parameters.

INPUT			
Geometric Dimension of the Test Piece		Slicer Parameters	
Parameter	Unit	Parameter	Unit
height	mm	Nozzle dimension	mm
lenght	mm	Line width	mm
width	mm	layer height	mm
		Number of outer lines	-
		α (raster angle)	°

**Table 2:** Algorithm main output parameters.

OUTPUT		
Parameter	Unit	Value—About Overall Part Volume
Volume of defects A	mm^3^	% of defects A
Volume of defects B	mm^3^	% of defects B
Volume of defects C	mm^3^	% of defects C
Volume of defects D	mm^3^	% of defects D
Total volume of defects	mm^3^	% total of defects

## Data Availability

The data presented in this study are available on request from the corresponding author.
